# Role of follistatin-like 1 levels and functions in calcific aortic stenosis

**DOI:** 10.3389/fcvm.2022.1050310

**Published:** 2023-01-06

**Authors:** Qianru Zhang, Jiawen Ye, Gan Yang, Ling Yang, Zhongli Chen, Ke Yang, Jia Teng Sun, Yan Liu

**Affiliations:** ^1^Department of Cardiology, Shanghai Ninth People’s Hospital, Shanghai Jiao Tong University School of Medicine, Shanghai, China; ^2^Department of Cardiology, School of Medicine, Renji Hospital, Shanghai Jiao Tong University, Shanghai, China; ^3^State Key Laboratory of Cardiovascular Disease, Arrhythmia Center, National Center for Cardiovascular Diseases, Fuwai Hospital, Chinese Academy of Medical Sciences and Peking Union Medical College, Beijing, China; ^4^Department of Cardiovascular Medicine, Ruijin Hospital, Shanghai Jiao Tong University School of Medicine, Shanghai, China

**Keywords:** calcific aortic stenosis, follistatin-like 1, calcification, valvular interstitial cell, calcific aortic valve disease

## Abstract

**Background:**

Calcific aortic valve disease (CAVD) is a progressive disease resulting in severe calcific aortic stenosis (AS), and there is increasing interest in the discovery of novel biomarkers to identify patients with potential future calcific AS at an early stage. This study aimed to determine whether follistatin-like 1 (FSTL1) is associated with calcific AS events and its exact role in aortic valve calcification.

**Methods:**

A prospective observational cohort study involving 656 patients was performed to investigate the relationship between serum FSTL1 and calcific AS incidence during a follow-up of 5 years. Furthermore, we detected FSTL1 levels in valvular interstitial cells (VICs) from calcified valves and explored the effects of FSTL1 on VIC osteogenic differentiation *in vitro* as well as the signaling pathways involved.

**Results:**

During a median follow-up of 5 years, lower FSTL1 levels were associated with a significantly higher risk of calcific AS events (log rank test, *P* = 0.007). In addition, Cox multivariable regression analyses verified the predictive value of FSTL1 after adjusting for both demographic features and laboratory confounders. Consistent with our results for serum, a lower concentration of FSTL1 was observed in calcified human valves (*n* = 11) and mainly colocalized with VICs. Recombinant human FSTL1 (rhFSTL1) stimulation inhibited calcium deposition, alkaline phosphatase (ALP) activity, and osteogenic gene expression partly through the downregulation of the ERK1/2 pathway.

**Conclusion:**

Taken together, this study provides a strong rationale to consider FSTL1 as a potential therapeutic target for calcific AS.

## Introduction

Calcific aortic valve disease (CAVD) is the most prevalent valvular heart disease worldwide (1, 2). CAVD is initiated by aortic valve sclerosis (AVSc) and progresses to calcific aortic stenosis (AS) at a rate of 2% every year (3). AVSc is the asymptomatic stage of CAVD and is characterized by the echocardiography finding of non-uniform thickening of the aortic leaflets without motive restriction. Several features are observed in AVSc, such as endothelial dysfunction, inflammation, lipid deposition and altered calcium metabolism (4, 5). Subsequent enhanced calcification and fibrosis within the aortic valve progress to calcific AS followed by restriction of blood flow and left ventricular remodeling (6, 7). However, determining whether patients are at high risk of the rapid progression to calcific AS or the ultimate stage of aortic-valve replacement is challenging. Therefore, the discovery of novel biomarkers to identify patients with potential future calcific AS at an early stage is of particular interest, and it will help us gain greater insight into the pathological mechanism of the disease.

Follistatin-like 1 (FSTL1), also known as follistatin-related protein (FRP), follistatin-like1 (FSL1), and TGF-beta-stimulated clone 36 (Tsc36), is an extracellular glycoprotein that belongs to the follistatin family and is highly conserved across species (8). Initial studies on FSTL1 have mainly focused on models of cancer and arthritis (9, 10). However, recent studies in several murine models suggest that FSTL1 also participates in the pathophysiology of cardiac disease. For example, FSTL1 overexpression minimizes myocardial apoptosis and ischemia–reperfusion injury, reduces postinfarct cardiac rupture, and modulates cardiomyocyte hypertrophy (11–16). Similarly, FSTL1 promotes endothelial function and revascularization in response to ischemic insult (17). During cardiac development, FSTL1 from atrioventricular valves plays a vital role in valve maturation and disease (18). Nevertheless, there is little information regarding the exact role of FSTL1 in aortic valve calcification.

A prospective observational cohort study was therefore performed to investigate the association between serum FSTL1 levels and calcific AS incidence during a follow-up of 5 years. Patients were divided into two groups based on the median FSTL1 level. We also determined the FSTL1 levels in valvular interstitial cells (VICs) from calcified valves and further explored whether FSTL1 regulates VIC osteogenic differentiation *in vitro* and its potential molecular mechanism.

## Materials and methods

### Study design and population

**FIGURE 1:**
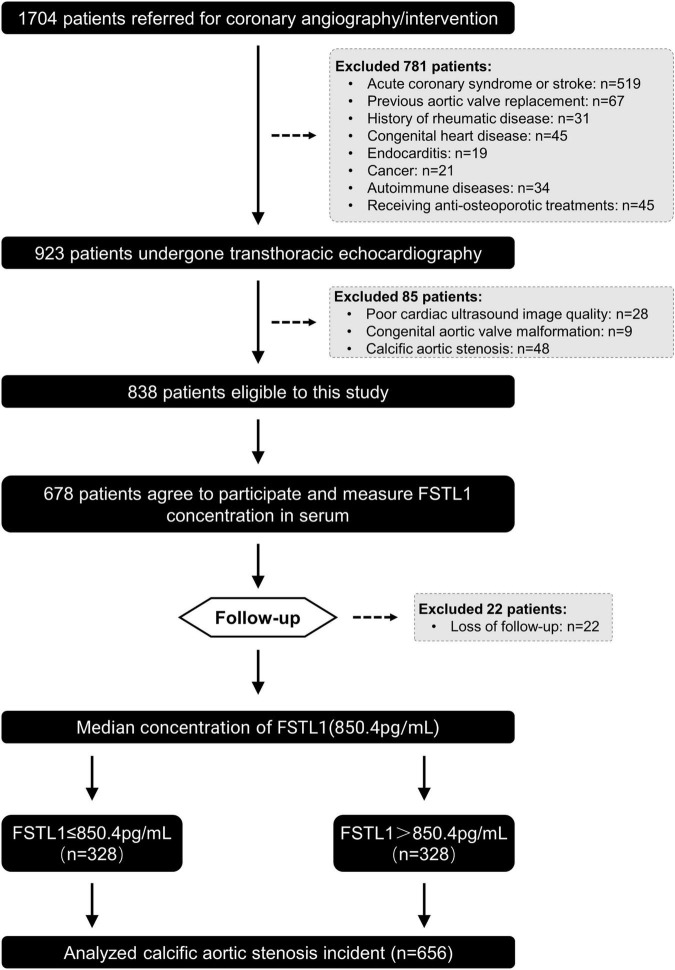
Flowchart of patient enrollment.

This was a prospective and observational cohort study. A total of 1,676 consecutive patients who underwent coronary angiography/intervention due to typical angina symptoms and/or electrocardiographic ST-T wave changes in Shanghai Rui Jin Hospital from January 2016 to December 2016 were enrolled. Patients with acute coronary syndrome or stroke (*n* = 519), previous aortic valve replacement (*n* = 67), rheumatic disease (*n* = 31), congenital heart disease (*n* = 45), endocarditis (*n* = 19), cancer (*n* = 21), autoimmune disease (*n* = 34) and receiving anti-osteoporotic treatment (*n* = 45) were excluded to avoid confounding influences. All the remaining 895 participants underwent standard two-dimensional echocardiography and Doppler flow imaging according to the recommendations of the American Society of Echocardiography (19). After echocardiographic examination, we further excluded patients with poor cardiac ultrasound image quality (*n* = 28), congenital aortic valve malformation (*n* = 9), and calcific AS (*n* = 48) and those who refused subsequent follow-up visits (*n* = 160). In total, 678 participants were invited for follow-up between 2016 and 2021. After excluding those who were lost to follow-up (*n* = 22), 656 participants were ultimately included in the analysis of the association between FSTL1 and calcific AS incidence (Figure 1). The primary outcome of this study was calcific AS. Data on the demographics, clinical, laboratory and angiographic features, and in-hospital management were collected. This study was conducted in accordance with the Declaration of Helsinki and approved by the regional ethics committee (Shanghai Jiao Tong University Ethics Committee). All patients provided written informed consent. Human calcified aortic valves were obtained from patients undergoing aortic valve replacement surgery during follow-up in our cohort (*n* = 11). Healthy aortic valves were collected from patients who had undergone heart transplantation (*n* = 12). The grouping criteria were consistent with the above.

### Echocardiographic evaluation

Transthoracic echocardiography was performed on all patients using a standard echocardiographic procedure at enrollment and during planned clinical assessments each year. The M-mode, 2-dimensional, and color Doppler echocardiographic analysis was conducted by an experienced echocardiographer. AVSc was defined as non-transparent aortic valve cusps with focal areas of mild thickening or increased echogenicity without motive restriction and a peak flow velocity across the aortic valve < 2.0 m/s (20). The presence of AS was defined as an aortic valve area of less than 2.0 cm^2^ (21). Calcific AS was confirmed upon the visualization of a thickened aortic valve with a restricted opening and peak aortic velocity > 2.0 m/s/mean gradient > 10 mmHg/aortic valve area < 2.0 cm^2^ (7).

### Assessment of serum FSTL1 concentrations

The ELISA kit used for FSTL1 was supported by Abcam (Cat# ab213782). Blood samples were collected from each participant before cardiac catheterization. Serum samples were processed according to standard procedures and then stored at −80°C. The concentration of FSTL1 was tested by using a commercial ELISA kit. Briefly, serum samples were added to each anti-FSTL1-coated well and then incubated with an anti-human FSTL1 primary antibody. After several washes, the streptavidin-enzyme conjugate was added to the well for detection. Finally, the level of bound antibody in each sample was quantified at 450 nm using a spectrophotometer. In this study, we measured the standard curve repeatedly and compared the intra- and interassay coefficients of variability, which were < 15% (CV%). The standard curves and the intra/inter assay data of the ELISA kit are shown in Supplementary Table 1.

### Reagents and antibodies

Primary antibodies against FSTL1 (Cat# ab71548), BMP2 (Cat# ab14933), and Msx2 (Cat# ab223692) and ELISA kits against FSTL1 (Cat# ab213782) were obtained from Cell Biolabs Abcam (Abcam, UK). The neutralization antibody against rhFSTL1 (Cat# HPAB-0165-WJ) was obtained from Creative Biolabs (Creative Biolabs, USA). Mouse IgG (Cat# sc-2025) was obtained from Santa Cruz Biotechnology (Santa Cruz Biotechnology, USA). Alizarin red S (Cat# A5533), cetylpyridinium chloride (Cat# 1104006), dexamethasone (Cat# D1756), L-ascorbic acid (Cat# 795437), β-glycerol phosphate (Cat# 50020), and collagenase II (Cat# C6885) were purchased from Sigma–Aldrich (Sigma–Aldrich, USA). Fetal bovine serum (FBS, Cat# 0500, ScienCell, USA), 1:1 DMEM:F12 culture medium (Cat# 11330057, Gibco BRL, USA), penicillin–streptomycin (Cat# 15140122, Gibco BRL, USA), and antibiotic-antimycotic solution (Cat# 15240062, Gibco BRL, USA) were used to culture cells. Cell Signaling Technology (USA) provided primary antibodies targeting GAPDH (Cat# 5174S), Vimentin (Cat# 5741S), and Runx2 (Cat# 8486S) as well as HRP-conjugated anti-mouse (Cat# 7074S) and HRP-conjugated anti-rabbit (Cat#7076S) secondary antibodies. Alexa Fluor 594-conjugated (Cat# R37119) or Alexa Fluor 488-conjugated (Cat#A27034) anti-rabbit antibodies obtained from Thermo Fisher Scientific (USA) were used for immunofluorescence staining. The Reverse Transcription System (Cat# A3500, Promega, USA) and SYBR^®^ Premix Ex Taq™ II (Tli RNaseH Plus, Cat# RR820A, Takara Bio, Japan) were used to measure mRNA levels. The forward and reverse primer sequences used were as follows: BMP2, 5′-GGG ACC CGC TGT CTT CTA GT-3′ and 5′-TCA ACT CAA ATT CGC TGA GGA C-3′; Runx2, 5′-AGT CCC AAC TTC CTG TGC T-3′ and 5′-GGT GAA ACT CTT GCC TCG TC-3′; Msx2, 5′-TTC ACC ACA TCC CAG CTT CTA-3′ and 5′-TTG CAG TCT TTT CGC CTT AGC-3′; and GAPDH, 5′-AGG TCG GTG TGA ACG GAT TTG-3′ and 5′-TGT AGA CCA TGT AGT TGA GGT CA-3′. An alkaline phosphatase (ALP) color development kit and a colorimetric kit were purchased from Beyotime Biotechnology (Cat# C3206 and Cat# P0321, Beyotime Biotechnology, China). The lentivirus was produced by Shanghai Genechem Company (Cat# 25317, Genechem, China).

### Immunofluorescence

Saline (0.9% NaCl) was used to wash residual blood cells from human aortic valve tissues. Then, the human aortic valve tissues were fixed with 4% paraformaldehyde, embedded in paraffin, and sectioned at a thickness of 5 μm. Masson staining was used to assess the degree of fibrosis of valves. Immunofluorescence staining of FSTL1 in human aortic valve tissue sections was performed using anti-FSTL1 antibodies at a dilution factor of 1:200, and anti-vimentin at a dilution of 1:200 was used to label valve interstitial cells (VICs). Then, the sectioned tissues were incubated with Alexa Fluor 594- or Alexa Fluor 488-conjugated anti-rabbit antibodies (1:500). All sections were tested on a machine after mounting with DAPI-containing mounting agent and observed with a laser confocal microscope (Zeiss 880).

### Isolation, culture, and treatment of primary mouse valve interstitial cells

Male mice (C57BL/6N background) were purchased from Charles River Company (China). Four-week-old mice were sacrificed, and the hearts were harvested and placed in washing buffer (PBS + 1,000 U/mL penicillin + 1,000 μg/mL streptomycin + 2.5 μg/mL amphotericin). Then, the aortic valves were isolated from the hearts under a stereoscope with an established microsurgical technique. The isolated leaflets were then cut into 1–2 mm^2^ pieces. These pieces were digested in 2 mg/mL collagenase II at 4°C for 12 h. This was followed by filtration and centrifugation. The cell pellets were resuspended and cultured (37°C and 5% CO2) in DMEM:F12 (1:1) containing 20% fetal bovine serum, penicillin (100 U/mL), and streptomycin (100 μg/mL). Cells between passages 2 and 3 were used for the study. Osteogenic medium containing DMEM supplemented with 10% FBS, 10 nM dexamethasone, 50 μg/mL ascorbate-2-phosphate, and 10 mM β-glycerol phosphate was used to induce VIC calcification. VICs were treated with rhFSTL1 (0 ng/mL, 25 ng/mL, 50 ng/Ml, or 100 ng/mL) for 7, 14, or 21 days. The medium was changed every 2 days.

### ALP staining and activity measurement

A commercial ALP Color Development Kit and a colorimetric kit were used to observe the formation of BNT-formazan and quantify the level of p-nitrophenol in VICs, respectively. ALP activity staining was viewed by using Image-Pro Plus 6.0. The absorbance of p-nitrophenol was read at 405 nm, and values were then normalized to a standard curve.

### Alizarin red staining

VICs were fixed with 4% paraformaldehyde for 25 min at room temperature followed by several washes with distilled water. Then, 2% Alizarin red S (pH 4.1) was used to stain VICs for 0.5 h at room temperature. For the quantitative analysis of Alizarin red S staining, cetylpyridinium chloride was incubated with the stained VICs for 15 min to release the dye from the cell matrix. The amount of released dye was quantified by spectrophotometry at 540 nm.

### Western blot analysis

Extracted proteins were solubilized in SDS-sample buffer, heated, subjected to SDS–PAGE, and then transferred to PVDF membranes. The membranes were incubated overnight at 4°C with primary antibodies against Bmp2 (1:500), Runx2 (1:1,000), Msx2 (1:1,000), or GAPDH (1:2,000). After several washes with TBST, the membranes were incubated with peroxidase-conjugated secondary antibodies (1:5,000) for 1 h at room temperature. Signals were detected with enhanced chemiluminescence (ECL) reagent.

### Statistical analysis

Continuous variables are presented as the mean with standard deviation (SD) when the data were normally distributed. If the data were not normally distributed, they are summarized as the median with interquartile ranges (IQRs). Patients were stratified according to the FSTL1 median levels. Categorical data are expressed as proportions and frequencies. Independent Student’s *t*-tests or non-parametric tests (Mann–Whitney *U*-Test) for continuous variables were used to compare differences between groups. Chi-squared tests were performed to compare categorical variables between the two groups. Calcific AS events among groups with different FSTL1 concentrations were compared by Kaplan–Meier analysis using the log-rank test. The relationship between FSTL1 serum level and clinical outcomes during follow-up was explored by uni- and multivariable Cox proportional hazard analyses with a 95% confidence interval (CI). The variables entered in Model 1 were age and sex. The variables entered in Model 2 were age, sex, hypertension, calcium, Lp (a) and eGFR. Directed acyclic graphs are shown in Supplementary Figure 1. The effect of different concentrations and treatment durations of FSTL1 on VIC calcification was tested by two-way ANOVA followed by the Bonferroni posttest in GraphPad Prism (v9.3.1). The remaining statistical tests were conducted with SPSS (v24.0) and R software (v7.2.0). A 2-sided *p*-value of less than 0.05 was considered to indicate a statistically significant difference.

## Results

### Baseline characteristics among patients with higher and lower FSTL1 levels

The baseline characteristics of the cohort are shown in Table 1. Increased fasting glucose and serum phosphorus levels were observed in patients with FSTL1 levels ≤ 850.4 pg/mL compared with those with higher FSTL1 levels; TG levels displayed the opposite trend. There was no significant difference in terms of age, sex, alcohol use, smoking or medical therapy between the two groups. The prevalence of diabetes, hypertension, AVSc and other biochemical biomarkers was comparable between the two groups. The baseline characteristics of patients who failed to be followed up are shown in Supplementary Table 2.

**TABLE 1:** Baseline characteristics of the study population.

	All patients (*n* = 656)	FSTL1 ≤ 850.4 pg/mL (*n* = 328)	FSTL1 > 850.4 pg/mL (*n* = 328)	*p*-value
Age, years	69 (62–75)	70 (64–76)	67 (60–73)	0.676
Male, *n* (%)	401 (61.1%)	210 (32.0%)	191 (29.1%)	0.128
BMI, kg/m^2^	24.46 (22.67–26.77)	25.16 (23.08–27.41)	24.00 (22.34–25.88)	0.878
Drinking, *n* (%)	83 (12.7%)	46 (14.0%)	37 (11.3%)	0.291
Smoking, *n* (%)	179 (27.3%)	95 (29.0%)	84 (25.6%)	0.335
Hypertension, *n* (%)	480 (73.2%)	244 (74.4%)	236 (72.0%)	0.481
CAD, *n* (%)	552 (84.1%)	279 (85.1%)	273 (83.2%)	0.521
DM, *n* (%)	197 (30.0%)	92 (28.0%)	105 (32.0%)	0.268
HbA1c,%	5.90 (5.60–6.58)	6.00 (5.60–6.70)	5.8 (5.56–6.40)	0.106
Fasting glucose, mmol/L	5.08 (4.58–5.90)	5.10 (4.61–6.03)	5.05 (4.57–5.71)	0.041
TG, mmol/L	1.32 (0.97–1.93)	1.27 (0.94–1.90)	1.38 (1.00–1.97)	0.014
TC, mmol/L	3.88 (3.18–4.57)	3.87 (3.16–4.57)	3.88 (3.25–4.60)	0.642
HDL-C, mmol/L	1.06 (0.91–1.25)	1.05 (0.90–1.21)	1.07 (0.92–1.29)	0.092
LDL-C, mmol/L	2.17 (1.67–2.84)	2.15 (1.68–2.81)	2.20 (1.68–2.86)	0.723
Lp (a), g/L	0.14 (0.08–0.29)	0.16 (0.09–0.41)	0.12 (0.07–0.23)	0.792
BUN, mmol/L	5.60 (4.60–6.60)	5.60 (4.7–6.8)	5.5 (4.6–6.3)	0.153
Scr, μmol/L	79.0 (69.0–91.0)	81.0 (69.5–93.0)	77.0 (66.0–88.0)	0.756
eGFR, mL/min/1.73 m^2^	76.98 (67.28–92.74)	76.36 (65.13–88.97)	85.08 (70.24–95.85)	0.752
γ-GT, IU/L	19 (14–28)	19 (14–28)	19 (13–29)	0.701
Ca, mmol/L	2.20 (2.13–2.28)	2.21 (2.13–2.28)	2.19 (2.12–2.26)	0.031
P, mmol/L	1.13 ± 0.18	1.15 ± 0.18	1.11 ± 0.18	0.003
Statins, *n* (%)	565 (86.1%)	284 (86.6%)	281 (85.7%)	0.735
Antidiabetic therapy, *n* (%)	108 (16.5%)	52 (15.9%)	56 (17.1%)	0.674
AVSc, *n* (%)	339 (51.7%)	162 (49.4%)	177 (54.0%)	0.241
FSTL1, pg/mL	850.40 (412.38–1376.87)	798.69 (343.17–1278.57)	899.90 (520.23–1491.42)	<0.001

Normally distributed variables: mean ± SD; skewed variables: median (interquartile range); categorical variable: *n* (%). For continuous variables, independent Student’s *t*-tests and Mann-Whitney *U*-tests were performed to assess differences. Differences in proportions were analyzed by 2 × 2 chi-square tests. BMI, body mass index; CAD, coronary artery disease; DM, diabetes; HbA1c: glycated hemoglobin; TG, triglyceride; TC, total cholesterol; HDL-C, high-density lipoprotein cholesterol; LDL-C, low-density lipoprotein cholesterol; Lp (a), lipoprotein a; BUN, blood urea nitrogen; Scr, serum creatinine; eGFR, estimated glomerular filtration rate; γ-GT, gamma-glutamyl transferase; FSTL1, follistatin-like 1; AVSc, aortic valve sclerosis.

### Influence of FSTL1 on calcific AS incidence

**FIGURE 2:**
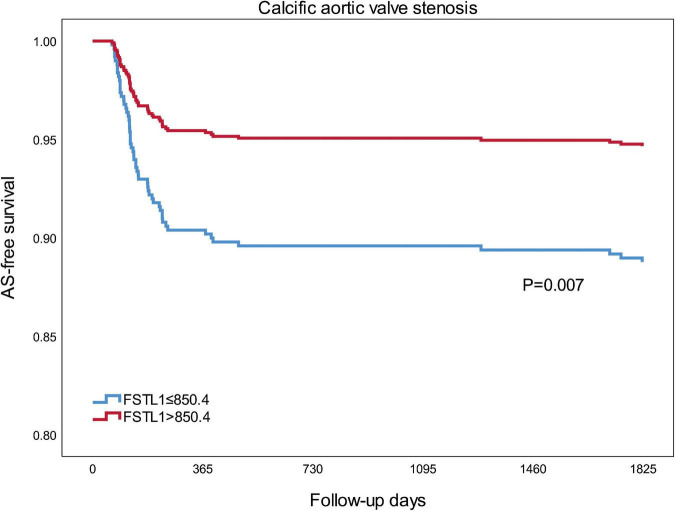
Kaplan-Meier curves for calcific aortic valve stenosis of two groups that were established using the median FSTL1 level. Differences among groups were evaluated with the log-rank test. FSTL1, follistatin-like 1; AS, aortic stenosis.

During a follow-up of 5 years, 56 (8.26%) incidents of calcific AS were documented, 38 (11.21%) in the lower FSTL1 group and 18 (5.31%) in the higher FSTL1 group. Kaplan–Meier survival analysis revealed that compared with higher FSTL1 levels, lower FSTL1 levels were associated with a significantly higher risk of calcific AS events (log rank test, *P* = 0.007) (Figure 2).

Furthermore, Cox regression analysis was performed to confirm the predictive effect of FSTL1 in the whole cohort and different subgroups. In either the unadjusted or adjusted model, lower FSTL1 levels indicated a significantly increased risk of calcific AS events in the whole cohort. Specifically, the hazard ratio for the prediction of calcific AS events in the overall cohort during follow-up was 0.462 (95% CI, 0.264–0.810, *P* = 0.007). The prognostic value of FSTL1 remained after adjustment for either demographic features alone (Model 1: HR, 0.468; 95% CI, 0.267–0.822, *P* = 0.008) or demographic features and laboratory confounders (Model 2: HR, 0.460; 95% CI, 0.261–0.809, *P* = 0.007).

**FIGURE 3:**
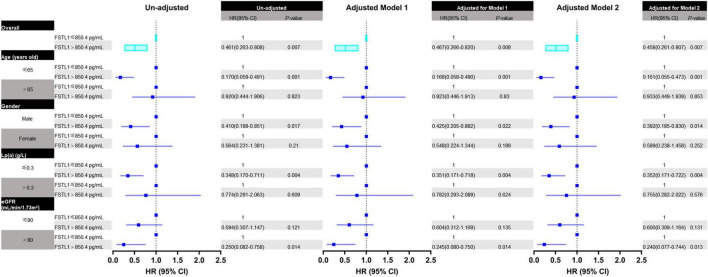
Forest plot for the association between FSTL1 levels and calcific aortic valve stenosis in all patients and different subgroups. The association between FSTL1 levels and calcific aortic valve stenosis was analyzed by uni- and multivariable Cox proportional hazard analyses. Log-rank tests were used to compare the impact of FSTL1 on patients. Variables entered in Model 1: age and sex. Variables entered in Model 2: age, sex, hypertension, calcium, Lp (a) and eGFR.

Among subgroup analyses, FSTL1 was associated with calcific AS events in male patients (HR, 0.413; 95% CI, 0.199–0.857, *P* = 0.018), patients with younger age (HR, 0.171; 95% CI, 0.059–0.494, *P* = 0.001), patients with decreased lipoprotein a [Lp (a)] levels (HR, 0.349; 95% CI, 0.171–0.714, *P* = 0.004), and patients without eGFR decline (HR, 0.250; 95% CI, 0.082–0.759, *P* = 0.014) in the univariable Cox model. Moreover, the predictive value of FSTL1 was still significant in the multivariable Cox model (Figure 3).

### Decreased FSTL1 levels are present in human calcific aortic valve lesions

**FIGURE 4:**
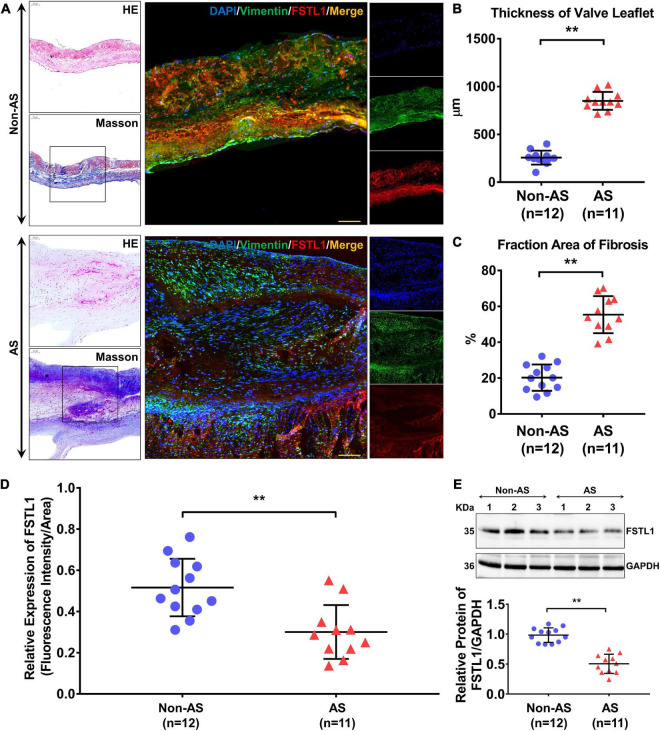
Protein levels of FSTL1 in aortic valve samples from non-AS (*n* = 12) and AS (*n* = 11) patients. **(A)** H&E and Masson staining were used to determine the thickness and degree of fibrosis of the aortic valve. Immunofluorescence was used to examine the protein levels and localization of FSTL1 in the aortic valve using vimentin as a marker of VICs. **(B)** The thickness of the aortic valve leaflet was measured with ImageJ software. **p < 0.01 vs. non-AS by Student’s *t*-test. **(C)** Fibrosis of aortic valves was calculated by the percentage of Masson staining percentage tissues. **p < 0.01 vs. non-AS by Student’s *t*-test. **(D)** Levels of FSTL1 compared between the two groups. **p < 0.01 vs. non-AS by Student’s *t*-test. **(E)** Western blotting was used to evaluate the expression level of FSTL1 in AS (*n* = 11) and non-AS (*n* = 12) aortic valve samples. **p < 0.01 vs. non-AS by Student’s *t*-test.

The expression level of FSTL1 in aortic valves was analyzed in 11 subjects with and 12 subjects without (i.e., non-AS subjects) calcific AS. The table of baseline characteristics of all the patients in both groups is shown in Table 2. In subjects with calcific AS, immunostaining demonstrated that the aortic valve leaflets were thicker (Figures 4A, B) and displayed more fibrosis and extracellular matrix remodeling than the leaflets in subjects without calcific AS (Figures 4A, C). Furthermore, in calcific AS subjects, there was a 42% reduction in FSTL1 levels around VICs (Figures 4A, D) (*p* < 0.01). We also detected the FSTL1 protein level in aortic valve samples by western blotting. The results showed that the level of FSTL1 in aortic valves of calcific AS was significantly decreased compared to that in non-AS valves (Figure 4E).

**TABLE 2:** Baseline characteristics of patients from whom aortic valves were obtained.

	All patients (*n* = 23)	non-AS (*n* = 12)	AS (*n* = 11)	*p*-value
Age, years	66 ± 8	61 ± 7	71 ± 5	<0.001
Male, *n* (%)	20 (87.0%)	10 (83.3%)	10 (90.9%)	1.000
BMI, kg/m^2^	21.41 ± 1.80	22.24 ± 1.93	20.51 ± 1.17	0.018
Drinking, *n* (%)	4 (17.4%)	1 (8.3%)	3 (27.3%)	0.317
Smoking, *n* (%)	5 (21.7%)	2 (16.7%)	3 (27.3%)	0.640
Hypertension, *n* (%)	15 (65.2%)	8 (66.7%)	7 (63.6%)	1.000
CAD, *n* (%)	23 (100.0%)	12 (100.0%)	11 (100.0%)	1.000
DM, *n* (%)	3 (13.0%)	3 (25.0%)	0 (0.0%)	0.217
HbA1c,%	5.78 ± 0.54	6.00 ± 0.47	5.52 ± 0.52	0.030
Fasting glucose, mmol/L	5.25 ± 0.61	5.30 ± 0.82	5.20 ± 0.28	0.684
TG, mmol/L	1.26 (0.90–1.60)	1.40 (0.94–2.06)	1.15 (0.89–1.35)	0.347
TC, mmol/L	4.45 ± 1.06	4.32 ± 0.92	4.59 ± 1.23	0.542
HDL-C, mmol/L	1.00 ± 0.12	0.95 ± 0.13	1.06 ± 0.09	0.036
LDL-C, mmol/L	2.82 ± 0.80	2.82 ± 0.78	2.83 ± 0.85	0.975
Lp (a), g/L	0.11 (0.05–0.49)	0.07 (0.04–0.63)	0.20 (0.07–0.39)	0.26
BUN, mmol/L	6.10 (5.00–7.70)	6.15 (4.68–7.38)	6.10 (5.60–8.40)	0.608
Scr, μmol/L	80.39 ± 22.94	86.83 ± 25.07	73.36 ± 19.02	0.164
eGFR, mL/min/1.73 m^2^	84.15 ± 17.76	83.27 ± 22.03	85.12 ± 12.56	0.809
γ-GT, IU/L	30.48 ± 12.46	32.17 ± 14.28	28.64 ± 10.51	0.510
Ca, mmol/L	2.25 ± 0.13	2.24 ± 0.16	2.26 ± 0.08	0.793
P, mmol/L	1.07 ± 0.24	1.18 ± 0.16	0.94 ± 0.27	0.017
Statins, *n* (%)	5 (21.7%)	4 (33.3%)	1 (9.1%)	0.317
Antidiabetic therapy, *n* (%)	3 (13.0%)	3 (25.0%)	0 (0.0%)	0.217

Normally distributed variables: mean ± SD; skewed variables: median (interquartile range); categorical variable: *n* (%). For continuous variables, independent Student’s *t*-tests and Mann-Whitney *U*-tests were performed to assess differences. Differences in proportions were analyzed by 2 × 2 chi-square tests. Abbreviations as in Table 1.

### FSTL1 reduces valve interstitial cell mineralization *in vitro*

**FIGURE 5:**
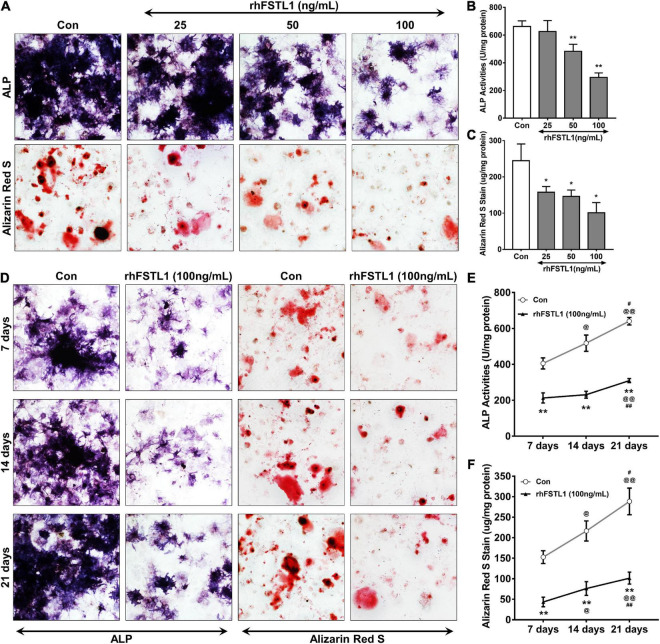
Effect of different concentrations and durations of FSTL1 treatment on the inhibition of mouse VIC calcification. **(A)** Different doses (0, 25, 50, and 100 ng/mL) of rhFSTL1 were added to the osteogenic medium for 21 days, and ALP activity and calcium deposition were measured with ALP and Alizarin Red S staining. **(B)** Quantification of ALP activity was determined by measuring the level of p-nitrophenol in mouse VICs. Data are from 3 independent experiments with 3 technical replicates. **p < 0.01 vs. Con by Student’s *t*-test. **(C)** Quantitative analysis of Alizarin red S staining was carried out by incubating stained mouse VICs with cetylpyridinium chloride. Data are from 3 independent experiments with 3 technical replicates. *p < 0.05 vs. Con by Student’s *t*-test. **(D)** rhFSTL1 (100 ng/mL) was added to the osteogenic medium for 7, 14, or 21 days. ALP activity and calcium deposition were measured by ALP and Alizarin Red S staining, respectively. **(E)** Quantification of ALP activity was performed by testing the level of p-nitrophenol in mouse VICs. Data are from 3 independent experiments with 3 technical replicates. **p < 0.01 vs. Con; ^@^*p* < 0.05, ^@@^*p* < 0.01 vs. 7 days; and ^#^*p* < 0.05, ^##^*p* < 0.01 vs. 14 days by two-way ANOVA followed by Bonferroni posttest. **(F)** Quantitative analysis of Alizarin red S staining was determined by incubating stained VICs with cetylpyridinium chloride. Data are from 3 independent experiments with 3 technical replicates. The control group was not treated with rhFSTL1. **p < 0.01 vs. Con; ^@^*p* < 0.05, ^@@^*p* < 0.01 vs. 7 days; and ^#^*p* < 0.05, ^##^*p* < 0.01 vs. 14 days by two-way ANOVA followed by Bonferroni posttest.

We utilized primary aortic VICs of mice cultured with osteogenic medium to mimic the process of mineralization. Different doses (0, 25, 50, and 100 ng/mL) of recombinant human FSTL1 (rhFSTL1) were added to the osteogenic medium to observe the effect of FSTL1 on the mineralization of VICs. After incubation with rhFSTL1 for 21 days, the mineralization of VICs decreased in a dose-dependent manner. In this context, 100 ng/mL FSTL1 exhibited the greatest inhibitory effect on calcification in VICs (Figures 5A–C). To verify whether FSTL1 delays VIC calcification, 100 ng/mL rhFSTL1 was added to the calcification induction medium, and the cells were incubated for 7, 14, and 21 days. The level of VIC calcification increased rapidly but was reduced by rhFSTL1 (Figures 5D–F). Collectively, the data show that the inhibitory effects of FSTL1 on VIC calcification were dose- and time-dependent.

### FSTL1 regulates osteogenic expression in VICs

**FIGURE 6:**
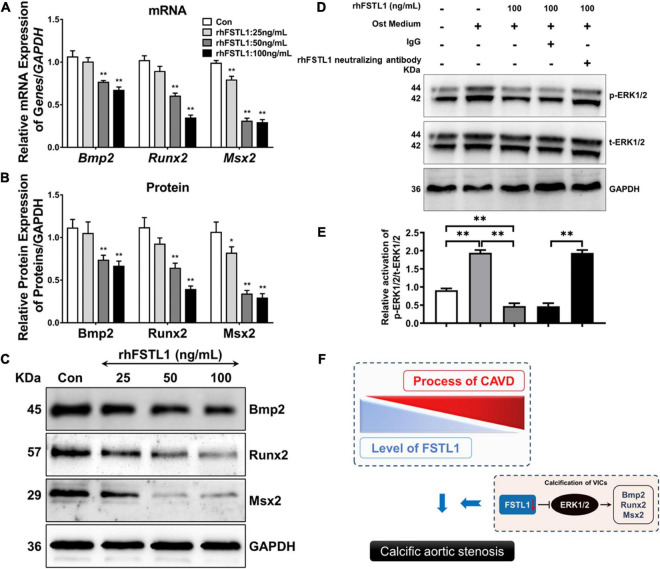
Effect of FSTL1 on the expression of osteogenic genes in VICs. **(A)** Different doses (0, 25, 50, and 100 ng/mL) of rhFSTL1 were added to the osteogenic medium for 7 days, and the mRNA levels of Bmp2, Runx2, and Msx2 were measured by RT–PCR. Data are from 3 independent experiments with 3 technical replicates. **p < 0.01 vs. Con by Student’s *t*-test. **(B)** Comparison of osteogenic gene expression levels. Data are from 3 independent experiments with 3 technical replicates. *p < 0.05, **p < 0.01 vs. Con by Student’s *t*-test. **(C)** Different doses (0, 25, 50, and 100 ng/mL) of rhFSTL1 were added to the calcification induction medium for 7 days, and the protein levels of Bmp2, Runx2, and Msx2 were measured by western blotting. **(D)** VICs were cultured in the presence or absence of rhFSTL1 (100 ng/mL) and treated with IgG or FSTL1 neutralizing antibody with osteogenic medium for 30 min. Western blotting was used to evaluate the level of phosphorylated ERK1/2. **(E)** Comparison of ERK1/2 phosphorylation levels. Data are from 3 independent experiments with 3 technical replicates. **p < 0.01 vs. Con by two-way ANOVA followed by Bonferroni posttest. **(F)** Schematic diagram showing that a reduction in FSTL1 expression leads to phosphorylation of ERK1/2, which then promotes the expression of osteogenic markers and eventually accelerates the progression of calcification. The control group was not treated with rhFSTL1.

The osteoblastic transition of VICs is the key link among the various pathological mechanisms of CAVD. Thus, we incubated primary VICs with rhFSTL1 for 7 days to determine whether FSTL1 regulates the mRNA and protein levels of osteogenic-related markers, including Bmp2, Runx2, and Msx2. rhFSLT1 significantly decreased the expression of Bmp2, Runx2, and Msx2, which suggests that FSTL1 is able to suppress calcification by downregulating osteogenic expression in VICs (Figures 6A–C). Then, we explored which pathway may be involved in the regulatory effects of FSTL1 on the expression of osteogenic-related markers. We observed that p-ERK1/2 levels were increased by stimulation with osteogenic medium. However, the level of p-ERK1/2 in the group pretreated with rhFSTL1 was significantly decreased. In addition, compared with the IgG group, the FSTL1 neutralizing antibody promoted the activation of ERK1/2 (Figures 6D, E).

## Discussion

To the best of our knowledge, this is the first study to highlight the beneficial impact of FSTL1 inhibiting calcification of the aortic valve. This conclusion was based on the following observations: (1) lower serum FSTL1 levels were predictive of calcific AS events, (2) decreased FSTL1 protein expression in human VICs was associated with valve calcification, and (3) FSTL1 inhibited osteoblastic differentiation of VICs partly through the downregulation of the ERK1/2 pathway. Collectively, our data provide evidence for a relationship between FSTL1 and calcific AS and implicate its anti-calcific role in the pathogenesis of the disease.

There are limited published data on the identification of biomarkers that are closely related to calcific AS incidence or progression, and these studies require a large-scale cohort with long-term follow-up. However, owing to its high morbidity and mortality, a strong incentive exists to identify key early molecules that contribute to the development of this disease. The current study revealed that approximately 9% of patients developed calcific AS events during the 5-year follow-up, which was a substantially greater rate of progression than that in other studies with large cohorts (22, 23). One possible explanation is that approximately 50% of patients presented with AVSc at enrollment. Second, over 80% of patients in our cohort had concomitant angiography-confirmed CAD, and it is well known that calcific AS follows a pathophysiological mechanism similar to that of atherosclerosis (24). Therefore, it was not surprising that a high portion of patients had an onset of calcific AS within 1 year of follow-up in our study, since a majority of these patients already had AVSc or CAD at enrollment. Our research further found that based on the median FSTL1 concentration, the low FSTL1 group had a significantly higher rate of echocardiographically determined calcific AS incidence. Similar trends were observed when patients were divided into tertiles according to their serum FSTL1 levels (Supplementary Figure 2). Interestingly, no significant differences in age or other traditional risk factors were observed between patients with higher or lower FSTL1 concentrations, suggesting a predictive effect independent of conventional risk factors. In addition, Cox multivariable regression verified the predictive value of FSTL1 after adjusting for both demographic features and laboratory confounders.

Notably, the association between FSTL1 and calcific AS events was accentuated among male patients, younger patients and patients without traditional risk factors. However, its diagnostic value declined in elderly patients with other complications. This is consistent with a previous study that showed that Lp (a) was associated only with a faster progression of calcific AS in patients aged ≤ 57 years (25). This suggests that as patients age, other risk factors and pathologic mechanisms might drive and accelerate the course of calcific AS development. As a result, FSTL1, a genetic risk factor, lost part of its initial independent predictive value. In a previous study, TG levels were associated with calcific AS (26); however, patients in the high FSTL1 cohort were associated with high TG levels in our cohort. We speculated that FSTL1 might also partly attenuate the pro-calcification properties of lipid disorders. This is of particular interest, as no medical treatment shows beneficial effects on slowing or reversing CAVD progression, indicating overly late intervention as well as the importance of discovering biomarkers for early risk stratification.

The close association between serum FSTL1 levels and calcific AS progression increased our interest in exploring the contribution of FSTL1 to the process of calcific AS pathophysiology. Consistent with our serum results, FSTL1 was decreased in tissues of calcified aortic valves. Specifically, VICs in the calcification region of the aortic valve displayed reductions in FSTL1 expression. We speculate that the attenuation of FSTL1 is a marker of calcification burden and that its restoration would be an effective way to inhibit the osteogenic process. To this end, rhFSTL1 was incubated with primary aortic VICs cultured with osteogenic medium, as the transformation of VICs to an osteoblastic phenotype was an important transition from the initiation phase to the progression phase of CAVD (27). Our study suggested that rhFSTL1 treatment led to a significant reduction in OST medium-induced VIC calcification. In particular, rhFSTL1 strongly reduced the calcium content and ALP levels, which are important markers of VIC osteogenic transformation, in a dose- and time-dependent manner. These results were consistent with a previous finding that the deletion of FSTL1 from the endocardial lineage leads to mitral regurgitation and eventually left ventricular diastolic dysfunction (18). This finding, along with our work, expanded the well-known cardiovascular protective role of FSTL1 in valve disease. There might be a synergistic effect of FSTL1 on aortic and atrioventricular valves, culminating in fibro-calcific leaflet remodeling and, ultimately, heart failure. In addition, previous studies have shown that FSTL1 has a double-edged role in inflammation, which is known to be another important cause of CAVD (28, 29). Therefore, targeting FSTL1 may be useful in the treatment of CAVD through the modulation of the inflammatory response.

BMP2 plays an important role in arterial and aortic valve calcification by regulating cell osteogenic transformation *via* the upregulation of Runx2 and Msx2 expression (30). A previous study showed that BMP-2 is only present in stenotic aortic valves, mostly in areas associated with focal calcium deposits, but not in healthy valves (1, 31). Accordingly, we demonstrated that rhFSLT1 exposure triggers the downregulation of BMP2, Runx2, and Msx2 levels in a dose-dependent manner. Consistently, it has been described that FSTL1 inhibits BMP signaling to prevent the excessive proliferation of mitral valve cells (18). ERK1/2 is a powerful regulator of BMP2 activity during calcification (32, 33). In line with this, enhanced ERK1/2 phosphorylation was observed in VICs stimulated with osteogenic medium. Furthermore, FSTL1 inhibited calcification-induced ERK1/2 phosphorylation. This inhibitory effect was attenuated by the FSTL1 antibody. Miyabe et al. also reported that FSTL1 can inhibit PDGF-BB-induced ERK1/2 activation in HASMCs (34). Hence, we hypothesize that FSTL1 inhibits the expression of osteogenic markers in VICs, at least partly, by blocking ERK1/2 phosphorylation (Figure 6F).

### Limitations

However, this study also had some limitations. First, this was a single-center cohort study and was limited to patients with a high rate of CAD. Whether the predictive value of FSTL1 can be applied to the general population or other samples of patients needs to be validated by future multicenter studies. Second, ACS patients were excluded since severe myocardial ischemia and enhanced inflammation might significantly influence FSTL1 levels (35, 36). Therefore, whether the association between FSTL1 and calcific AS still remains in ACS needs further investigation. Third, we lacked *in vivo* results, and further animal experiments will be conducted to confirm the calcification inhibitory effects of FSTL1 on AS reported here. Fourth, in terms of *in vitro* studies, pro-calcific medium and human VICs should be used to validate our results.

## Conclusion

Lower serum FSTL1 levels at admission were found to predict the development of calcific AS events, mainly in patients with younger age or those without traditional risk factors. We also found decreased FSTL1 levels in the aortic valve, and rhFSTL1 treatment impeded the osteogenic responses of VICs *in vitro*. Therefore, this study provides a strong rationale for the consideration of FSTL1 as a potential therapeutic target for calcific AS.

## Data availability statement

The raw data supporting the conclusions of this article will be made available by the authors, without undue reservation.

## Ethics statement

The studies involving human participants were reviewed and approved by the Ruijin Hospital Ethics Committee, Shanghai Jiao Tong University School of Medicine. The patients/participants provided their written informed consent to participate in this study. The animal study was reviewed and approved by the Shanghai Ninth People’s Hospital, Shanghai Jiao Tong University School of Medicine. Written informed consent was obtained from the owners for the participation of their animals in this study.

## Author contributions

KY, JS, and YL provided the study design and interpretation, wrote and edited the manuscript, were the guarantors of this work, and took full responsibility for the work as a whole, including the study design, access to the data and decision to submit and publish the manuscript. QZ, JY, GY, LY, and ZC performed the data collection and analysis. QZ contributed to the interpretation of the data and the drafting and editing of the manuscript. All authors contributed to the conception and design, acquisition of the data or analysis and interpretation of the data, drafting the article or revising it critically for important intellectual content and gave final approval of the version to be published.

## Supplementary material

Supplementary Figure 1 Directed acyclic graph.

Supplementary Figure 2 Kaplan-Meier curves for calcific aortic valve stenosis divided into tertiles according to serum FSTL1 levels. Differences among groups were evaluated with the log-rank test. FSTL1, follistatin-like 1; AS, aortic stenosis.
