# Supplementary material for: Role of follistatin-like 1 levels and functions in calcific aortic stenosis
Source: Front Cardiovasc Med. 2023 Jan 6;9:1050310. doi: 10.3389/fcvm.2022.1050310 (PMC9852832; doi:10.3389/fcvm.2022.1050310)
Supplement: Supplementary file 1 [file Table_1.DOCX]

**Supplementary Table 1.** **The standard curves and the intra- and inter-assay data of the ELISA.**

|  | **standard Concentrations (pg/mL)** | **20000** | **10000** | **5000** | **2500** | **1250** | **625** | **312** | **0** |
| --- | --- | --- | --- | --- | --- | --- | --- | --- | --- |
| **Plate 1** | Dilutions OD 1 | 2.469 | 1.391 | 0.829 | 0.522 | 0.341 | 0.233 | 0.209 | 0.162 |
|  | Dilutions OD 2 | 2.440 | 1.300 | 0.809 | 0.502 | 0.327 | 0.249 | 0.208 | 0.161 |
|  | inter- assay CV% | 0.008 | 0.048 | 0.017 | 0.028 | 0.030 | 0.047 | 0.003 | 0.004 |
| **Plate 2** | Dilutions OD 1 | 2.610 | 1.610 | 0.959 | 0.600 | 0.367 | 0.280 | 0.238 | 0.189 |
|  | Dilutions OD 2 | 2.605 | 1.578 | 0.978 | 0.570 | 0.380 | 0.297 | 0.241 | 0.193 |
|  | inter- assay CV% | 0.001 | 0.014 | 0.014 | 0.036 | 0.025 | 0.042 | 0.009 | 0.015 |
| **Plate 3** | Dilutions OD 1 | 2.477 | 1.412 | 0.833 | 0.541 | 0.374 | 0.289 | 0.275 | 0.197 |
|  | Dilutions OD 2 | 2.462 | 1.354 | 0.817 | 0.532 | 0.344 | 0.260 | 0.258 | 0.201 |
|  | inter- assay CV% | 0.004 | 0.030 | 0.014 | 0.012 | 0.059 | 0.075 | 0.045 | 0.014 |
| **Plate 4** | Dilutions OD 1 | 2.543 | 1.591 | 0.704 | 0.445 | 0.267 | 0.288 | 0.230 | 0.171 |
|  | Dilutions OD 2 | 2.554 | 1.503 | 0.745 | 0.476 | 0.287 | 0.281 | 0.223 | 0.163 |
|  | inter- assay CV% | 0.003 | 0.040 | 0.040 | 0.048 | 0.051 | 0.017 | 0.022 | 0.034 |
| **Plate 5** | Dilutions OD 1 | 2.777 | 1.418 | 0.972 | 0.472 | 0.283 | 0.229 | 0.219 | 0.161 |
|  | Dilutions OD 2 | 2.536 | 1.418 | 0.815 | 0.452 | 0.257 | 0.230 | 0.199 | 0.175 |
|  | inter- assay CV% | 0.064 | 0.000 | 0.124 | 0.031 | 0.068 | 0.003 | 0.068 | 0.059 |
| **Plate 6** | Dilutions OD 1 | 2.424 | 1.496 | 0.894 | 0.643 | 0.390 | 0.290 | 0.221 | 0.180 |
|  | Dilutions OD 2 | 2.435 | 1.439 | 0.909 | 0.632 | 0.368 | 0.279 | 0.229 | 0.193 |
|  | inter- assay CV% | 0.003 | 0.027 | 0.012 | 0.012 | 0.041 | 0.027 | 0.025 | 0.049 |
| **Plate 7** | Dilutions OD 1 | 2.552 | 1.366 | 0.822 | 0.608 | 0.381 | 0.269 | 0.204 | 0.175 |
|  | Dilutions OD 2 | 2.540 | 1.343 | 0.788 | 0.620 | 0.354 | 0.266 | 0.203 | 0.173 |
|  | inter- assay CV% | 0.003 | 0.012 | 0.030 | 0.014 | 0.052 | 0.008 | 0.003 | 0.008 |
| **Plate 8** | Dilutions OD 1 | 2.491 | 1.411 | 0.818 | 0.632 | 0.443 | 0.244 | 0.167 | 0.138 |
|  | Dilutions OD 2 | 2.540 | 1.422 | 0.814 | 0.643 | 0.421 | 0.223 | 0.164 | 0.133 |
|  | inter- assay CV% | 0.014 | 0.005 | 0.003 | 0.012 | 0.036 | 0.064 | 0.013 | 0.026 |
| **Plate 9** | Dilutions OD 1 | 2.527 | 1.441 | 0.769 | 0.678 | 0.335 | 0.266 | 0.178 | 0.149 |
|  | Dilutions OD 2 | 2.524 | 1.386 | 0.739 | 0.667 | 0.343 | 0.248 | 0.179 | 0.139 |
|  | inter- assay CV% | 0.001 | 0.028 | 0.028 | 0.012 | 0.017 | 0.050 | 0.004 | 0.049 |
|  | intra- assay CV% | 0.033 | 0.060 | 0.095 | 0.144 | 0.145 | 0.091 | 0.141 | 0.122 |
