# Supplementary material for: Role of follistatin-like 1 levels and functions in calcific aortic stenosis
Source: Front Cardiovasc Med. 2023 Jan 6;9:1050310. doi: 10.3389/fcvm.2022.1050310 (PMC9852832; doi:10.3389/fcvm.2022.1050310)
Supplement: Supplementary file 2 [file Table_2.DOCX]

**Supplementary Table 2. Baseline characteristics of follow-up and loss of follow-up patients.**

|  | **Patients of follow-up (n=656)** | **Loss of follow-up(n=22)** | ***P*-value** |
| --- | --- | --- | --- |
| **Age, years** | 69.00(62.00-75.00) | 68(62.75-73.25) | 0.756 |
| **Male, n (%)** | 401(61.1%) | 13(59.1%) | 0.828 |
| **BMI, kg/m^2^** | 24.46(22.67-26.77) | 24.23(24.15-24.31) | 0.569 |
| **Drinking, n (%)** | 83(12.7%) | 3(13.6%) | 0.751 |
| **Smoking, n (%)** | 179(27.3%) | 6(27.3%) | 1.000 |
| **Hypertension, n (%)** | 480(73.2%) | 16(72.7%) | 1.000 |
| **CAD, n (%)** | 552(84.1%) | 19(86.4%) | 1.000 |
| **DM, n (%)** | 197(30.0%) | 6(28.6%) | 1.000 |
| **HbA1c, %** | 5.90(5.60-6.58) | 5.93(5.88-5.98) | 0.929 |
| **Fasting glucose, mmol/L** | 5.08(4.58-5.90) | 5.08(5.03-5.13) | 0.990 |
| **TG, mmol/L** | 1.32(0.97-1.93) | 1.33(1.11-1.86) | 0.667 |
| **TC, mmol/L** | 3.88(3.18-4.57) | 3.86(3.36-4.41) | 0.961 |
| **HDL-C, mmol/L** | 1.06(0.91-1.25) | 1.06(1.01-1.11) | 0.918 |
| **LDL-C, mmol/L** | 2.17(1.67-2.84) | 2.18(2.02-2.340 | 0.983 |
| **Lp(a), g/L** | 0.14(0.08-0.29) | 0.15(0.10-0.19) | 0.605 |
| **BUN, mmol/L** | 5.60(4.60-6.60) | 5.59(5.54-5.64) | 0.913 |
| **Scr, μmol/L** | 79.0(69.0-91.0) | 80.10(79.15-81.05) | 0.788 |
| **eGFR, mL/min/1.73m^2^** | 76.98(67.28-92.74) | 77.21(76.59-77.84) | 0.444 |
| **γ-GT, IU/L** | 19(14-28) | 19.00(13.75-24.25) | 0.512 |
| **Ca,mmol/L** | 2.20(2.13-2.28) | 2.21(2.16-2.26) | 0.675 |
| **P,mmol/L** | 1.13±0.18 | 1.13±0.06 | 0.978 |
| **Statins, n (%)** | 565(86.1%) | 19(86.4%) | 1.000 |
| **Antidiabetic therapy, n (%)** | 108(16.5%) | 4(18.2%) | 0.773 |
| **AVSc, n (%)** | 339(51.7%) | 11(50.0%) | 1.000 |
| **FSTL1, pg/mL** | 850.40(412.38-1376.87) | 840.87(402.56-1380.93) | 0.932 |

*Normally distributed variables: mean ± SD; skewed variables: median (interquartile range); categorical variable: n (%). For continuous variables, independent Student’s t tests and Mann-Whitney U Test* *were performed to assess differences. Differences in proportions were analyzed by 2×2 chi-square tests. BMI, body mass index; CAD, coronary artery disease; DM, diabetes; HbA1c: glycated hemoglobin; TG, triglyceride; TC, total cholesterol; HDL-C, high-density lipoprotein cholesterol; LDL-C, low-density lipoprotein cholesterol; Lp(a): lipoprotein a; BUN: blood urea nitrogen; Scr: serum creatinine; eGFR, estimated glomerular filtration rate; γ-GT, gamma-glutamyl transferase; FSTL1, follistatin-like 1; AVSc, aortic valve sclerosis.*
