# Supplementary figures and images for: Role of follistatin-like 1 levels and functions in calcific aortic stenosis
Source: Front Cardiovasc Med. 2023 Jan 6;9:1050310. doi: 10.3389/fcvm.2022.1050310 (PMC9852832; doi:10.3389/fcvm.2022.1050310)

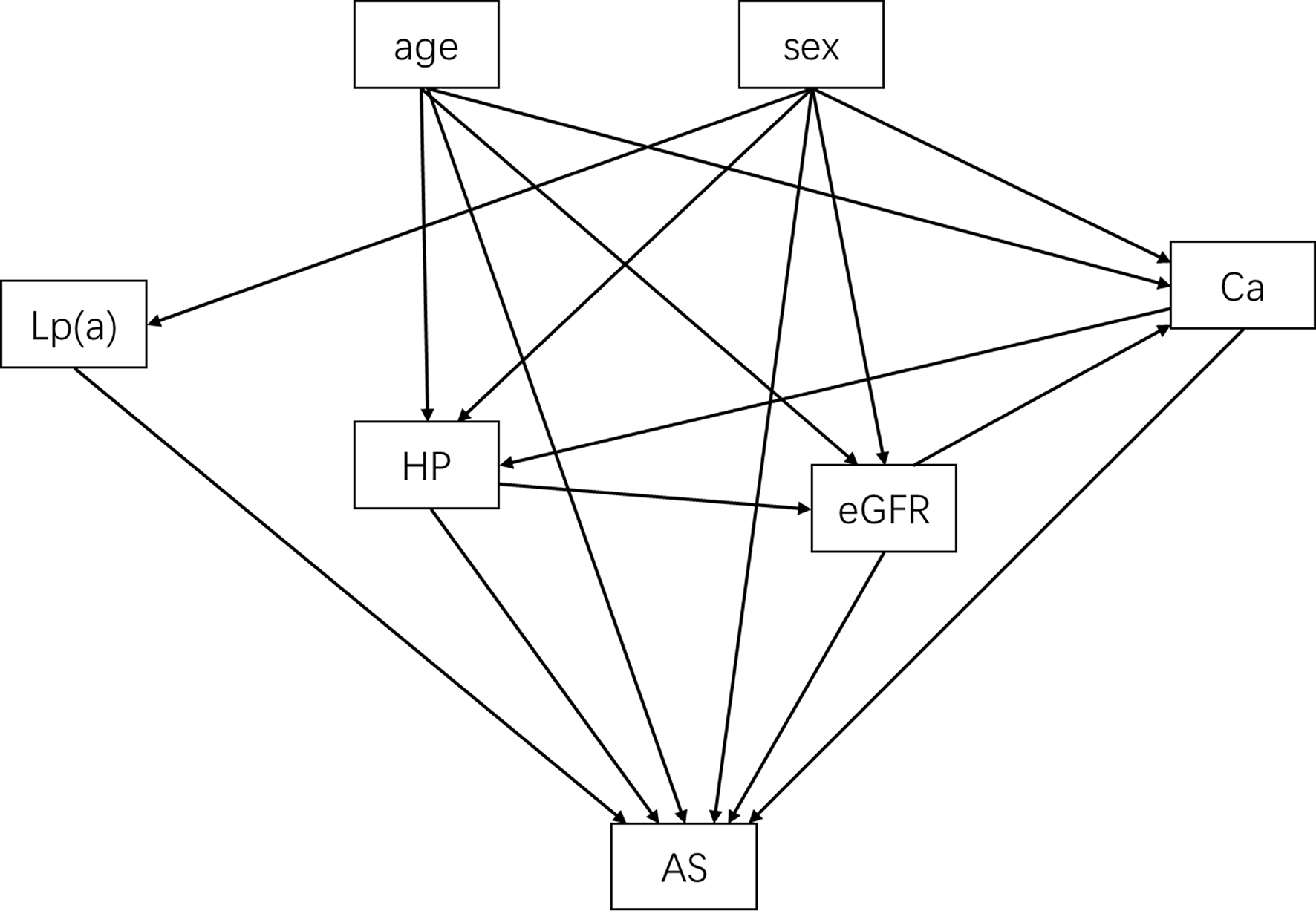

Supplement: Supplementary Figure 1 — Directed acyclic graph. [file Image_1.TIF]

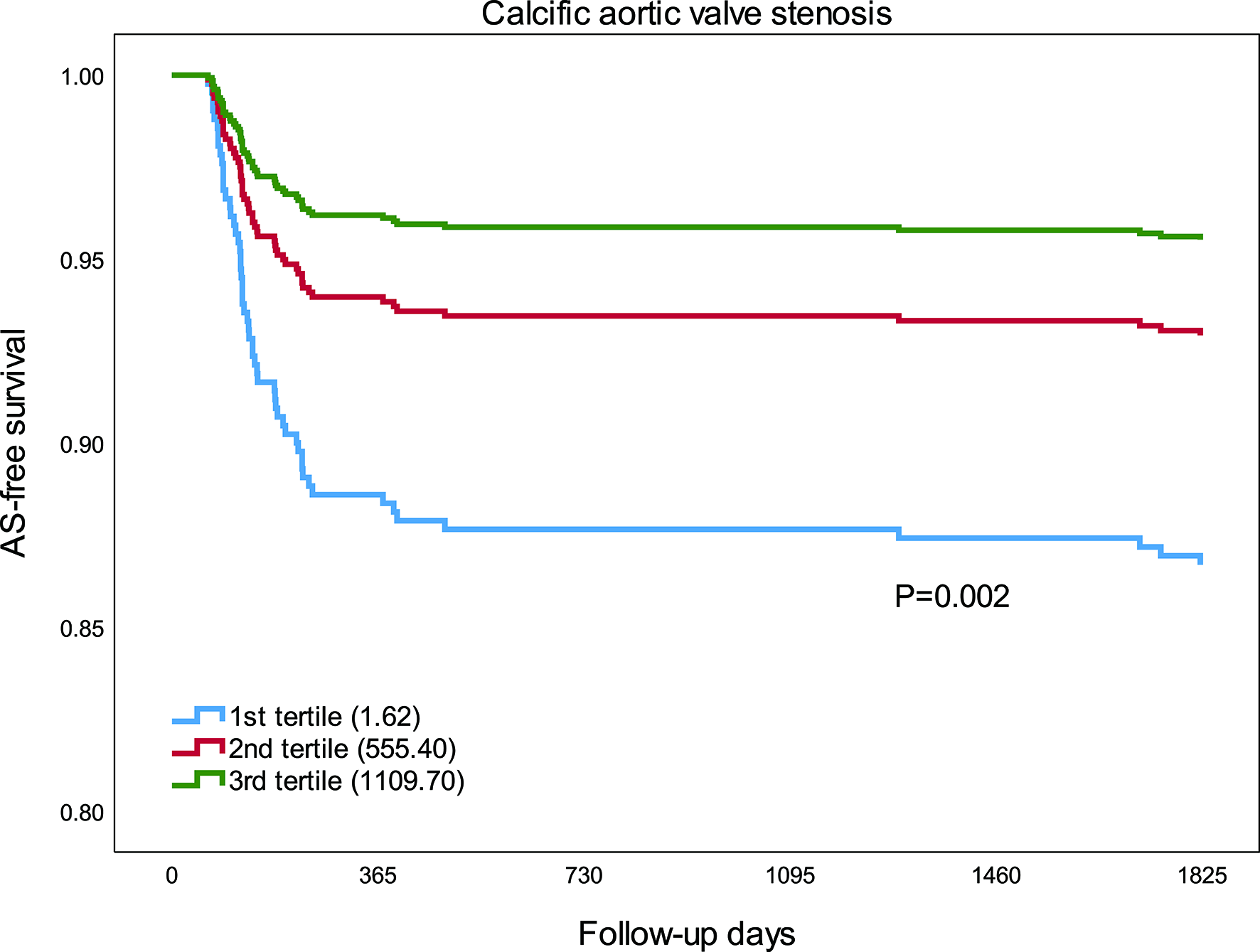

Supplement: Supplementary Figure 2 — Kaplan-Meier curves for calcific aortic valve stenosis divided into tertiles according to serum FSTL1 levels. Differences among groups were evaluated with the log-rank test. FSTL1, follistatin-like 1; AS, aortic stenosis. [file Image_2.TIF]
